# Dendritic Cell Activity Driven by Recombinant *Mycobacterium bovis* BCG Producing Human IL-18, in Healthy BCG Vaccinated Adults

**DOI:** 10.1155/2015/359153

**Published:** 2015-08-03

**Authors:** Piotr Szpakowski, Franck Biet, Camille Locht, Małgorzata Paszkiewicz, Wiesława Rudnicka, Magdalena Druszczyńska, Fabrice Allain, Marek Fol, Joël Pestel, Magdalena Kowalewicz-Kulbat

**Affiliations:** ^1^Department of Immunology and Infectious Biology, Institute of Microbiology, Biotechnology and Immunology, University of Lodz, Banacha Street 12/19, 90-237 Lodz, Poland; ^2^UMR1282, Infectiologie et Santé Publique (ISP-311), INRA-Centre Val de Loire, 37380 Nouzilly, France; ^3^Center for Infection and Immunity of Lille, Institut Pasteur de Lille, 59019 Lille, France; ^4^Inserm U1019, 59019 Lille, France; ^5^CNRS UMR 8204, 59019 Lille, France; ^6^Université Lille Nord de France, 59019 Lille, France; ^7^CNRS-UMR 8576, Unité de Glycobiologie Structurale et Fonctionnelle, IFR 147, Université Lille Nord de France, Université de Lille 1, 59655 Villeneuve d'Ascq, France

## Abstract

Tuberculosis remains an enormous global burden, despite wide vaccination coverage with the Bacillus Calmette-Guérin (BCG), the only vaccine available against this disease, indicating that BCG-driven immunity is insufficient to protect the human population against tuberculosis. In this study we constructed recombinant BCG producing human IL-18 (rBCGhIL-18) and investigated whether human IL-18 produced by rBCGhIL-18 modulates DC functions and enhances Th1 responses to mycobacterial antigens in humans. We found that the costimulatory CD86 and CD80 molecules were significantly upregulated on rBCGhIL-18-infected DCs, whereas the stimulation of DCs with nonrecombinant BCG was less effective. In contrast, both BCG strains decreased the DC-SIGN expression on human DCs. The rBCGhIL-18 increased IL-23, IL-10, and IP-10 production by DCs to a greater extent than nonrecombinant BCG. In a coculture system of CD4^+^ T cells and loaded DCs, rBCGhIL-18 favoured strong IFN-*γ* but also IL-10 production by naive T cells but not by memory T cells. This was much less the case for nonrecombinant BCG. Thus the expression of IL-18 by recombinant BCG increases IL-23, IP-10, and IL-10 expression by human DCs and enhances their ability to induce IFN-*γ* and IL-10 expression by naive T cells, without affecting the maturation phenotype of the DCs.

## 1. Introduction

The global burden of mycobacterial infections is still one of the major public health concerns, despite the widespread use of the Bacillus Calmette-Guérin (BCG) vaccine, which confers good protection against disseminated childhood tuberculosis (TB), but provides variable protection against pulmonary disease in adults.

There is good evidence that the protection obtained by BCG vaccination declines with age. Thus, improved vaccines are desperately needed, but their development is hampered by the insufficient knowledge of the immune protection mechanisms. One approach to develop a new TB vaccine is improving BCG by the expression of immune modulators for superior targeting of immune pathways, which are essential in protective immunity [1]. The Th-1 responses have long been recognized as essential mechanisms of protection against mycobacterial diseases, including tuberculosis [2, 3]. Interleukin 18, first described in 1989 as “IFN-*γ*-inducing factor," initially isolated from the serum of* M. bovis *BCG-infected mice challenged with LPS [4], is considered to play a significant role in promoting Th1 responses. It is produced by activated macrophages and dendritic cells (DCs) but also by T and B cells and many other cell types [5–7]. IL-18 can be a potent therapeutic tool against severe bacterial infections [8]. Moreover, it has been shown that systematic administration of IL-18 enhances the regression of well-established primary tumors by a mechanism that depends on CD8+  T cell, Fas/FasL, and endogenous IFN-*γ*, particularly in combination with other cytokines [9–11]. However, recent studies have shown that exogenous IL-18 given to mice induced an exaggerated inflammatory reaction, which led to adverse effects including neutrophil-mediated lung and/or intestinal injuries [12]. It may limit the potential use of IL-18 as a therapeutic agent in humans. To overcome this difficulty, we hypothesized that a bacterial construct producing a small amount of IL-18 could directly modulate the antigen presentation by DCs for the upregulation of Th1 responses, yet avoiding the potential harmful effects of exogenous IL-18. BCG bacilli, which are strong Th1 inducers, seem to be a useful platform for IL-18 expression. IL-18 has been shown to enhance the host defense against mycobacterial infections [13–15]. Mice deficient for IL-18, but not for the IL-18 receptor, are highly susceptible to* M. tuberculosis *infection [16]. In mice, BCG can synergize with IL-18 for IFN-*γ* production, potentially leading to enhanced protective immunity against mycobacteria [17]. The synergistic effect of IL-18 on IFN-*γ* production depends on the IL-18 receptors expressed on Th1 but not on Th2 cells [18]. It was observed that infection of mice with recombinant BCG strain producing murine IL-18 (rBCGmIL-18) strongly enhanced the ability of BCG to polarize the immune response towards a Th1 type [19]. In a mouse model of MBT-2 superficial bladder cancer, recombinant BCG secreting IL-18 also enhanced macrophage cytotoxicity against cancer cells in an IFN-*γ* dependent manner [20]. In a murine experimental allergic asthma model rBCGmIL18 was shown to be more efficient in suppressing allergen-driven pulmonary Th2 responses and eosinophilia than nonrecombinant BCG [21]. IL-18 also augments IFN-*γ* in human macrophages infected with* M. tuberculosis *[22]. However, still little is known about the combined effect of mycobacteria and IL-18 on human DCs.

In this study, we constructed a recombinant BCG strain producing human IL-18 (rBCGhIL-18) and examined its effects on human monocyte-derived DCs (MoDCs), obtained from healthy young subjects who had been immunized with BCG at birth and school age. In this human model we compared the effects of BCG and rBCGhIL-18 on MoDC taking into account: (a) the expression of cell-surface signaling receptors, which are engaged in the direct interaction between DCs and T cells, (b) the production of cytokines, which provide the differentiation of responding T cells, and (c) the efficient induction of antigen-specific effector functions of CD4^+^ T cells.

## 2. Materials and Methods

### 2.1. Construction of the Human IL-18 Expression Vector

**Figure 1:**
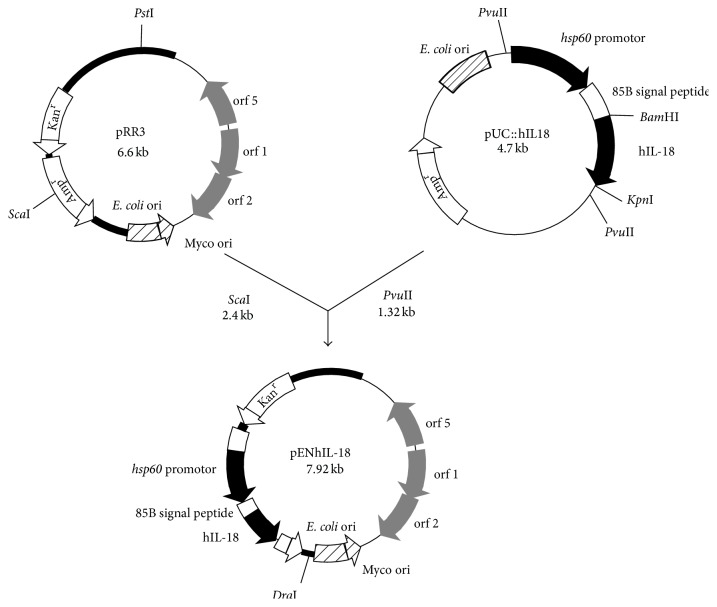
Construction of pENhIL-18 used to produce and secrete hIL-18 by rBCGhIL-18. White arrows Kan^R^ and Amp^R^ represent the kanamycin and ampicillin resistance genes, respectively. ColE1 represents the origin of replication from* E.coli* (hatched boxes), and oriM represents the mycobacterial origin of replication (hatched arrows). The expression cassette contains the BCG* hsp60 *promoter, the ribosome-binding site, and the* hsp60 *initiating codon represented by the black box. The* M. tuberculosis *alpha-antigen signal peptide coding sequence is represented by the white box, whereas the IL-18 coding sequence is shown in black. The grey boxes indicate open reading frames of the mycobacterial plasmid, necessary for replication in BCG.

The* M. bovis* BCG vaccine strain 1173P2 (World Health Organization, Stockholm, Sweden) was genetically modified to produce mature human IL-18 (hIL-18) by transformation with pENhIL-18 (Figure 1), a pRR3 derivative [23] containing the mature hIL-18-encoding gene under the control of the BCG* hsp60* promoter and modified by replacing the original signal peptide cleavage sequence with the mycobacterial signal peptide coding sequence from the BCG *α*-antigen. The 512 bp DNA fragment encoding hIL-18 was obtained by reverse transcription-PCR on total RNA from U-937 cells activated for 6 h with LPS (1 *μ*g/mL). Total RNA was extracted using RNAzol (Applied Oncor) according to the manufacturer's recommendation. Reverse transcription-PCR was carried out as described previously [17] with the gene-specific primers with the following sequences: 5′-TATAGGATCCTACTTTGGCAAGCTTGAA-3′ and 5′-TATAGGTACCGGCATGAAATTTTAATAGC-3′ (Eurogentec, Liège, Belgium). The 126-bp DNA fragment encoding the *α*-antigen signal sequence was amplified by PCR using chromosomal BCG DNA extracted as described previously [24] with the primers 5′- GGCACAGGTCATGACAGACGTGAGCCGAAAGATTCGA-3′ and 5′-GCCGGGATCCCGCGCCCGCGGTTGCCGCTCCGCC-3′ (Eurogentec). The PCR fragment encoding hIL-18 restricted by* Bgl*II and* Asp*718 and the PCR fragment encoding the *α*-antigen signal sequence restricted by* Bsp*HI and* Bam*HI were inserted into pUC::*hsp60 *[24] restricted by* Nco*I and* Asp*718, thereby generating pUC::hIL-18. The 1.32 kb* Pvu*II fragment from pUC::hIL-18 spanning the BCG* hsp60 *promoter, the ribosomal binding site, the *α*-antigen signal peptide coding sequence, and the mature hIL-18 coding sequence, was inserted into pRR3, previously digested with* Sca*I. The resulting shuttle vector, pENhIL-18, was used to transform BCG, and the transformants were selected by their resistance to Kanamycin. Kanamycin-resistant BCG colonies were analyzed for their plasmid content by using electroduction [25]. The recombinant strain was named rBCGhIL-18.

### 2.2. Detection of HIL-18 in Recombinant Mycobacteria

Mycobacterial cell extracts were prepared as described previously [19] from 10 mL cultures harvested at mid-log phase. The proteins in the lysates, corresponding to ca. 5 × 10^6^ bacteria, were separated by SDS-PAGE on a 15% polyacrylamide gel [26]. Total proteins were then transferred onto a Hybond-C extra membrane (Amersham France). The membrane was saturated with 1% bovine serum albumin in phosphate-buffered saline (PBS)—0.1% Tween 20 (PBST) and then incubated with rabbit anti-IL-18 antiserum diluted 1/2,000. Goat anti-rabbit alkaline phosphatase-conjugated antibodies (Promega, Madison, Wis. USA) diluted 1/7.000 in PBST were then used to develop the immunoblots.

### 2.3. Bacterial Strains and Growth Conditions

*M. bovis* BCG (Pasteur strain 117P2; WHO Stockholm, Sweden) and rBCGhIL-18 were grown to the mid-log phase in stationary flasks, at 37°C in 7H9 Middlebrook liquid medium (Becton Dickinson) supplemented with: 10% oleic acid-albumin-dextrose catalase (OADC) (Difco, BD Biosciences) and 0.05% Tween 80, and frozen until use.

For the colony forming units (CFU) counts, bacteria were serially diluted in PBS containing 0.05% Tween 80 and plated on Middlebrook 7H11 agar supplemented with 10% OADC enrichment. Bacteria were grown for 3-4 weeks at 37°C for CFU enumeration.

### 2.4. Blood Donors

Blood was collected from 60 young healthy volunteers aged 25–35, vaccinated with BCG in childhood, according to state policy. All studies were approved by the local Ethic Commitee. Healthy volunteers signed the consent for the participation in the study before blood collection.

### 2.5. Monocyte-Derived DC Preparation

Peripheral blood was obtained using vacutainer tubes with spray-coated heparin (Becton Dickinson). Monocytes were separated from PBMC by immunomagnetic positive separation using CD14^+^ Microbeads (Miltenyi Biotech, Germany) [27]. CD14^+^ cell purity was determined to be 96% to 99% on the basis of forward and side scatter gating in conjunction with CD14 staining using standard flow cytometry (data not shown).

Monocytes were suspended in RPMI-1640 (Sigma-Aldrich, Germany) supplemented with 100 U/mL penicillin, 0.1 mg/mL streptomycin and L-glutamine (Polfa Tarchomin, Poland) and enriched with 10% (v/v) fetal calf serum (FCS, heat inactivated; Cambrex, Belgium). The density was adjusted to 1 × 10^6^/mL, and the monocytes were placed into 6-well flat-bottomed culture plates and allowed to differentiate into DCs by incubation for 6 days in RPMI-1640 supplemented with 1% antibiotics and 10% FCS in the presence of 25 ng/mL human GM-CSF and 10 ng/mL human recombinant IL-4 (R&D Systems, USA). After 6 days of culture, the cells were harvested, pooled, and counted.

### 2.6. Stimulation of DC with BCG, rBCGhIL-18 or Purified Protein Derivative (PPD)

Immature DCs were placed into 6-well plates at a density of 1 × 10^6^ cells/mL and incubated for 24 h (37°C, 5% CO_2_) with live BCG, rBCGhIL-18 at a multiplicity of infection (MOI) of 1 : 1 or with 10 *μ*g/mL PPD (Statens Serum Institut, Copenhagen, Denmark). Lipopolysaccharide (LPS) from* Escherichia coli* O55:B5 (1 *μ*g/mL) (Sigma) was used as positive control of DC activity and DC in medium alone (unpulsed DC) represented the negative control. To assess whether neutralizing anti-human IL-18 antibodies reduce the effect of rBCGhIL-18 on activating cells, mouse monoclonal anti-human IL-18 antibody (MBL) (1 *μ*g/mL) was added to the culture of unstimulated and stimulated DC.

### 2.7. DCs Preparation for Flow Cytometry

The antigen-stimulated and unstimulated DCs were collected from the 6-well plate by using PBS/2 mM EDTA. After washing in PBS the DC were incubated for 30 min at 4°C with the following mAb: fluorescein isothiocyanate (FITC)-conjugated anti-CD86, anti-CD40, anti-HLA-DR, anti-DC-SIGN; phycoerythrin (PE)-conjugated anti-CD80, or an irrelevant isotype-matched mAb used as control. All mAbs were purchased from Becton Dickinson (BD). Data were acquired and analyzed using the FACS LSRII (BD) and FlowJo software, a minimum 10,000 events were collected. The calculated mean fluorescence intensity represents the molecule density on the cell surface and the percentage of positive DC for each marker.

### 2.8. Cytokine and Chemokine Concentration

Supernatants of 1 × 10^6^ purified DCs, pulsed or not with BCG, rBCGhIL-18, PPD, or LPS, were harvested 24 h following stimulation, centrifuged (400 ×g for 10 min), and stored at −20°C until further used. Supernatants were then tested by the ELISA, (Eli-pair Diaclone test) for the presence of  IL-10, IL-12p70, and IL-23 (detection sensitivity: 5 pg/mL for IL-10 and IL-12p70; 20 pg/mL for IL-23), human IL-18 (RayBiotech: detection sensitivity 0.5 pg/mL) and for the presence of  IP-10 (CXCL10) by using specific ELISA (R&D systems: detection sensitivity: 5 pg/mL). The cytokines were quantified by reference to a standard curve obtained for individual cytokine standards provided by the manufacturers.

### 2.9. T Cell Preparation

Autologous naive CD45RA^+^CD4^+^ and memory CD45RO^+^CD4^+^ cells were isolated from the eluted CD14^−^ cell fraction by using a naive CD4^+^ T-cell isolation kit, as previously described [28], or the memory CD4^+^ T-cell isolation kit (Miltenyi), according to the manufacturer's protocol. Both isolated cell fractions (purity > 95%) were frozen until use.

### 2.10. DC-T Cell Coculture

The preserved naive and memory T lymphocytes 1 × 10^7^ cells/mL were thawed and cocultured with BCG-, rBCGhIL-18-,or PPD- (10 *μ*g/mL) primed DC at a ratio of 10 T cells per one infected DC, for 5 days at 37°C with 5% CO_2_. Collected supernatants were tested for IFN-*γ* and IL-10 production by ELISA using commercially available kits (Diaclone). The limit of detection was 5 pg/mL for both IFN-*γ* and IL-10.

### 2.11. Statistical Analysis

All data were analyzed using the STATISTICA 8.0 PL software. Differences between antigens were evaluated for each parameter using nonparametric Kruskal-Wallis test. When statistical significance was observed, differences were analyzed by Mann-Whitney *U* test (for impaired data) to verify the hypothesis that two analyzed samples came from two statistically different populations. *P* values < 0.05 were considered significant.

## 3. Results

### 3.1. Production and Secretion of hIL-18 by rBCGhIL-18

**Figure 2:**
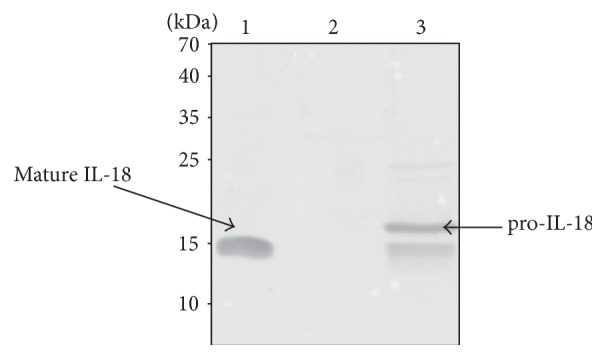
Immunoblot analysis of rBCGhIL-18. Lane 1 contains 100 ng of mature IL-18. Whole-cell extracts of nonrecombinant BCG (lane 2) or rBCGhIL-18 (lane 3), each corresponding to an optical density at 600 nm of 0.2 were subjected to SDS-PAGE and analyzed by immunoblotting using rabbit anti-IL-18 antibodies. The sizes of the molecular weight markers are indicated in the left margin, and the presence of mature IL-18 and pro-IL-18 is indicated by arrows.

To construct rBCGhIL-18, the hIL-18-encoding cDNA was prepared as described previously [19] and modified by substituting the original IL-18 signal peptide coding sequence with the mycobacterial secretion signal sequence from the BCG *α*-antigen [29]. The modified hIL-18 cDNA was inserted into the expression vector pRR3 [23] (Figure 1) and then introduced into BCG. The recombinant BCG construction, named rBCGhIL-18, was found to produce human IL-18, as shown by immunoblot analyses using an anti-human IL-18 rabbit antiserum. Immunoreactive proteins were detected in the lysate of rBCG producing hIL-18, but not in the lysate of the nonrecombinant control strain (Figure 2). The rBCGhIL-18 extract contained an immune-reactive protein with a size expected for noncleaved pro-IL-18, as well as bands whose *M*_*r*_ are similar to that of mature IL-18, indicating that the pro-hIL-18 in rBCGhIL-18 is partially processed into the mature form, similarly to our previous study describing the expression and immunological activity of murine IL-18 produced by recombinant BCG [19]. The IL-18 expression by recombinant BCG was checked during the entire study period, always with the same results. The concentration of hIL-18 was quantified by ELISA in cell culture supernatants of BCG- or rBCGhIL-18-stimulated DC. For the three healthy individuals-tested, cultures contained 5–10 pg/mL and 18–22 pg/mL hIL-18, after 24 h stimulation with BCG or rBCGhIL-18, respectively. Unstimulated cultures contained only 3–7 pg/mL hIL-18.

### 3.2. Maturation Marker Expression by Mycobacteria-Pulsed DCs

**Figure 3:**
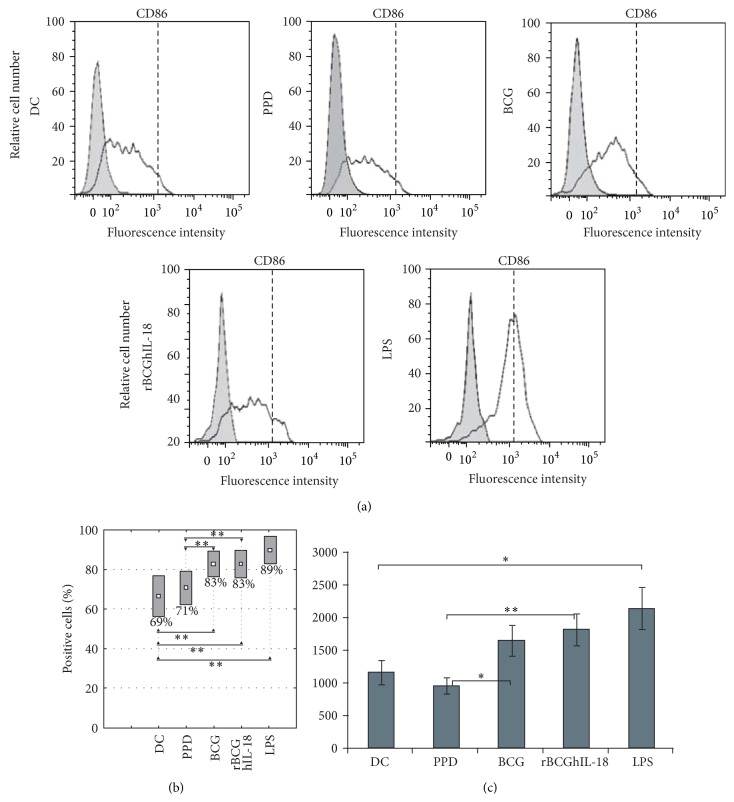
CD86 surface expression of MoDCs. Human MoDCs were pulsed either with PPD (10 *μ*g/mL), BCG (1 : 1), rBCGhIL-18 (1 : 1), or LPS (1 *μ*g/mL) for 24 h or were left unstimulated (DC). (a) One representative experiment out of 20 independent ones is shown. Grey histograms represent the cell reactivity to fluorochrome-matched isotype control antibodies. The white histograms represent the reactivity with the anti-CD86 antibody. The vertical broken lines represent the upregulation obtained by LPS stimulation; (b) percentage of positive cells with the CD86 expression; (c) median fluorescence intensity (MFI) values (median ± SEM of 20 independent donors). Fluorescence intensity was calculated by the MFI of the receptor expression from which the MFI obtained with a nonrelevant, isotype-matched antibody was substracted. Statistical analyses were performed using the Kruskal-Wallis test. ^*^ *P* < 0.05; ^**^ *P* < 0.01.

**Figure 4:**
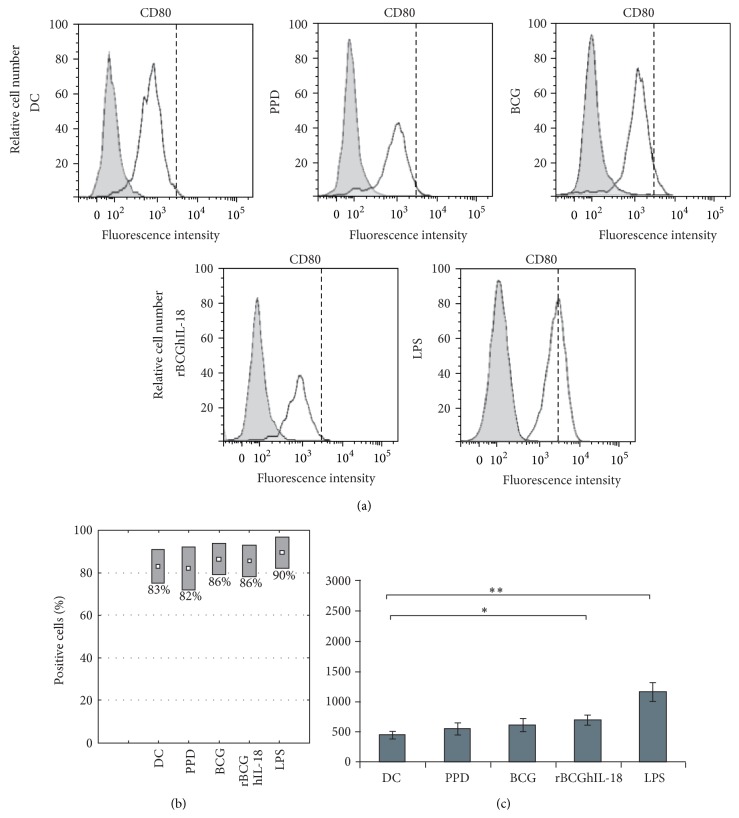
CD80 surface expression of MoDCs. Human MoDCs were pulsed either with PPD (10 *μ*g/mL), BCG (1 : 1), rBCGhIL-18 (1 : 1), or LPS (1 *μ*g/mL) for 24 h or were left unstimulated (DC). (a) One representative experiment out of 20 independent ones is shown. Grey histograms represent the cell reactivity to fluorochrome-matched isotype control antibodies. The white histograms represent the reactivity with the anti-CD80 antibody. The vertical broken lines represent the upregulation obtained by LPS stimulation; (b) percentage of positive cells with the CD80 expression; (c) median fluorescence intensity (MFI) values (median ± SEM of 20 independent donors). Fluorescence intensity was calculated by the MFI of the receptor expression from which the MFI obtained with a nonrelevant, isotype-matched antibody was substracted. Statistical analyses were performed using the Kruskal-Wallis test. ^*^ *P* < 0.05; ^**^ *P* < 0.01.

**Figure 5:**
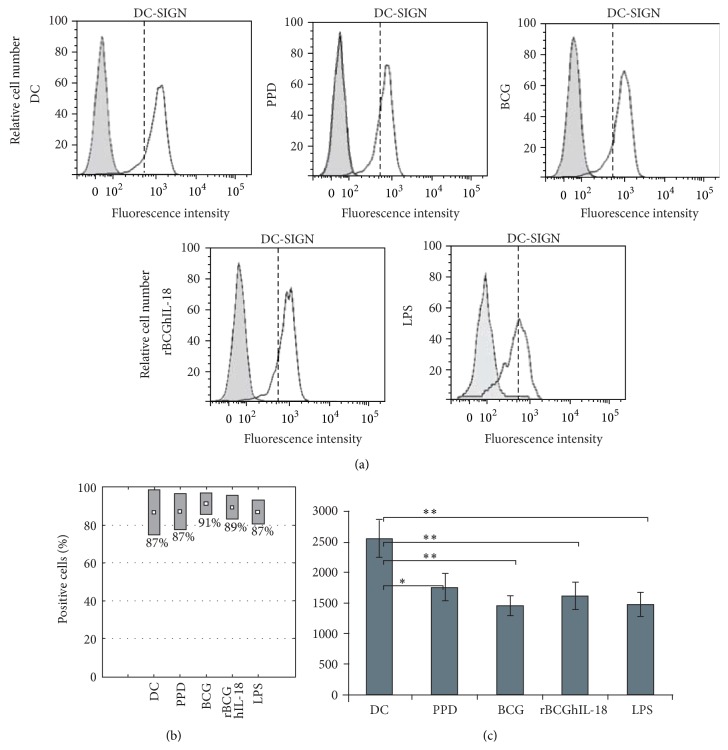
DC-SIGN surface expression of MoDCs. Human MoDCs were pulsed either with PPD (10 *μ*g/mL), BCG (1 : 1), rBCGhIL-18 (1 : 1), or LPS (1 *μ*g/mL) for 24 h or were left unstimulated (DC). (a) One representative experiment out of 20 independent ones is shown. Grey histograms represent the cell reactivity to fluorochrome-matched isotype control antibodies. The white histograms represent the reactivity with the anti-DC-SIGN antibody. The vertical broken lines represent the upregulation obtained by LPS stimulation; (b) percentage of positive cells with the DC-SIGN expression; (c) median fluorescence intensity (MFI) values (median ± SEM of 20 independent donors). Fluorescence intensity was calculated by the MFI of the receptor expression from which the MFI obtained with a nonrelevant, isotype-matched antibody was substracted. Statistical analyses were performed using the Kruskal-Wallis test. ^*^ *P* < 0.05; ^**^ *P* < 0.01.

To investigate the effect of rBCGIL-18 on human monocyte-derived DC maturation, the DCs were pulsed with rBCGhIL-18, BCG, or PPD and compared to control conditions (i.e., unstimulated DCs as a negative control, and LPS-pulsed DCs as a positive control). Various T cell costimulation surface markers (CD86, CD80, and CD40), as well as HLA-DR expression, were assessed by flow cytometry. LPS induced the upregulation of CD86 (Figure 3(a)) and CD80 (Figure 4(a)) as well as CD40 and HLA-DR (data not shown), and the downregulation of DC-SIGN. DC incubation with PPD did not modify the expression of CD86, CD80, CD40, and HLA-DR, but downregulated the expression of DC-SIGN. In contrast, the incubation with either of the two BCG strains at an MOI of 1 : 1 induced the upregulation of some of these surface markers and again the downregulation of DC-SIGN. Moreover, we could detect a higher proportion of BCG- and rBCGhIL-18-stimulated MoDCs with CD86 expression, as compared to unstimulated DC, and those incubated with PPD (Figure 3(b)). This difference was not observed in the case of CD80 (Figure 4(b)), HLA-DR, CD40 markers (data not shown) and DC-SIGN (Figure 5(a)). When the median of 20 different donors was analyzed, a significant increase in CD86 expression was observed upon incubation with BCG or with rBCGhIL-18 compared with untreated DC and those incubated with PPD (Figure 3(c)). In parallel, a significant decrease in DC-SIGN expression was observed upon incubation with BCG, rBCGhIL-18, PPD or LPS, consistent with a DC maturation phenotype. However, there was no significant difference between BCG and rBCGhIL-18. (Figure 5(c)).

### 3.3. Effect of BCG and rBCGhIL-18 on Cytokine and Chemokine Production by Human DCs

**Figure 6:**
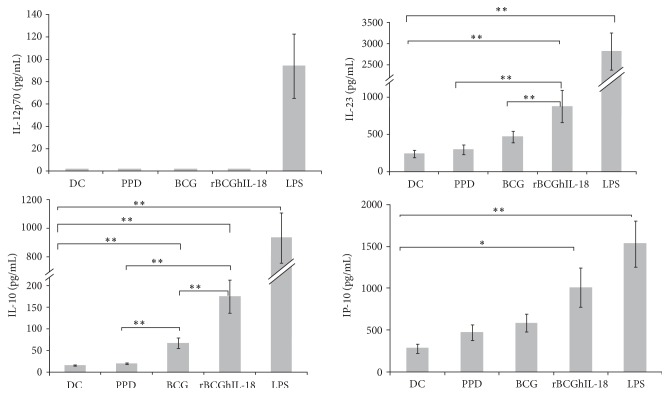
IL-12p70, IL-10, IL-23, and IP-10 production by stimulated MoDCs. Human MoDCs were stimulated with PPD (10 *μ*g/mL), BCG (1 : 1), rBCGIL-18 (1 : 1), or LPS (1 *μ*g/mL) for 24 h or were left untreated (DC). The cytokine and chemokine levels in the culture were measured by ELISA. Data shown are the mean ± SEM of 40 independent donors. Statistical analyses were performed using the Kruskal-Wallis test. ^*^ *P* < 0.05; ^**^ *P* < 0.01.

Next we analyzed the cytokine profile of the mycobacterial-pulsed DC that might affect the T-cell polarization. In particular, we analyzed IL-12, IL-23, and IL-10 in the supernatants of the DCs incubated with PPD, BCG, or rBCGhIL-18. In all blood donors, LPS-primed MoDCs produced significant levels of IL-12p70. In contrast, DCs infected with BCG and rBCGhIL-18 or stimulated with PPD, failed to secrete IL-12p70 in 6-hour cultures (data not shown) as well as in 24 hour cultures (Figure 6). Since bioactive IL-12 consists of p35 and p40 subunits, and the p40 subunit is also present in other IL-12 family members, such as IL-23, we determined the amount of secreted IL-23 in the supernatants of mycobacterial-stimulated DC cultures. Among the mycobacterial products, only rBCGhIL-18 induced significant IL-23 production compare to unpulsed DCs (*P* = 0.001), albeit much less than LPS (roughly 1/3 less). A trend was also seen for nonrecombinant BCG, but did not reach statistical significance, leading therefore to statistical significance between BCG and rBCGhIL-18 (Figure 6). In addition to IL-23, rBCGhIL-18 also induced the production of IL-10 by the DCs, although significant amounts of IL-10 were also produced upon stimulation with BCG, but not with PPD. However, rBCGhIL-18 induced significantly more IL-10 than BCG (*P* = 0.001), albeit again much less than LPS. Finally, rBCGhIL-18 also induced significant levels of IP-10 (CXCL10), known to be involved in Th1 cell recruitment, whereas this was not the case for PPD or nonrecombinant BCG, suggesting that rBCGhIL-18 might favor T cell towards a Th1 profile.

### 3.4. rBCGhIL-18 Induces IFN-*γ* and IL-10 Production by Naive T Cells rather than by Memory T Cells

**Figure 7:**
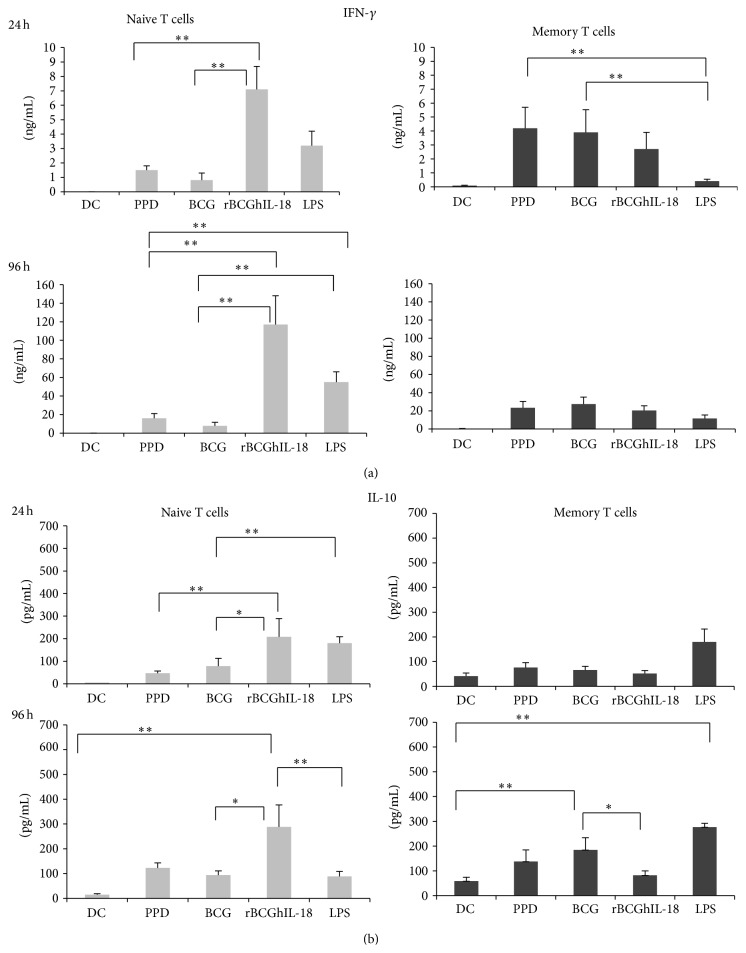
Secretion of IFN-*γ* (a) and IL-10 (b) by human naive (grey bars) and memory (black bars) T cells following 24 h and 96 h coculture with PPD- (10 *μ*g/mL), BCG- (1 : 1), rBCGIL-18- (1 : 1), or LPS- (1 *μ*g/mL) pulsed autologous MoDCs (ratio MoDCs/T cells, 1 : 10). The cytokine levels in the cocultures were measured by ELISA. Data shown are the mean ± SEM of 20 independent donors. Statistical analyses were performed using the Kruskal-Wallis test and Mann-Whitney *U* test. ^*^ *P* < 0.05; ^**^ *P* < 0.01.

In order to investigate whether rBCGhIL-18-pulsed DC can polarize T cells towards the Th1 profile, we analyzed the cytokine responses of the autologous naive and memory CD4^+^ T cells upon incubation with DCs stimulated with PPD, BCG, or rBCGhIL-18. As shown in Figure 7(a), naive T cells produced significantly more IFN-*γ* in response to rBCGhIL-18-stimulated DC coincubation, as compared to T cells incubation with DCs stimulated with PPD or BCG, both after 24 h and after 96 h of culture. PPD- or BCG-stimulated DCs induced only low levels of IFN-*γ* by the naive T cells. Similarly, rBCGhIL-18-pulsed DC also stimulated the secretion of IL-10 by naive T cells, whereas this was much less the case for PPD- or BCG-pulsed DCs (Figure 7(b)) In contrast to the naive T cells, memory T cells produced IFN-*γ* upon coculture with BCG-, rBCGhIL-18-, or PPD-pulsed DC at comparable levels. Similar results were obtained for the production of IL-10 by the T cells. Interestingly, both IFN-*γ* and IL-10 were produced at higher levels by naive T cells upon incubation with rBCGhIL-18-pulsed DC, compared to memory T, whereas the reverse was seen for IFN-*γ* upon coculture with PPD- or BCG-pulsed DCs. There were no differences in the IL-10 production by naive and memory T cells in response to PPD-pulsed MoDCs.

**Figure 8:**
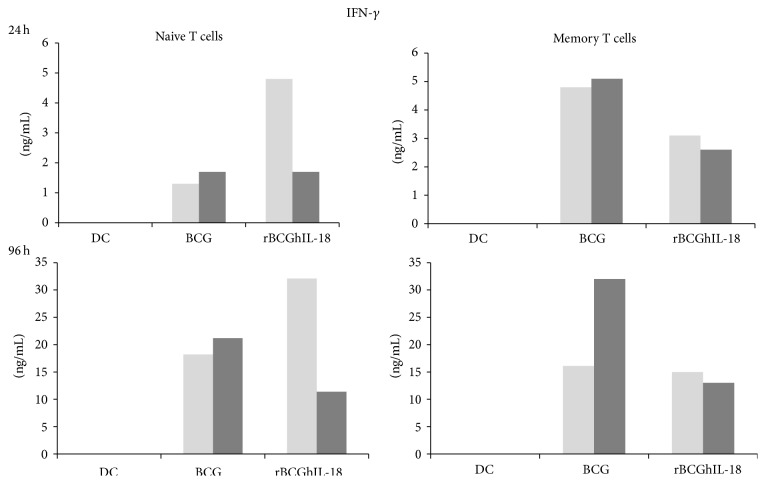
Secretion of  IFN-*γ* by human naive and memory T cells following 24 h and 96 h coculture with BCG- (1 : 1), rBCGIL-18- (1 : 1) pulsed autologous MoDCs (ratio MoDCs/T cells, 1 : 10) in the presence (black bars) of neutralizing human anti-IL-18 antibody or not (grey bars). The cytokine level in the cocultures was measured by ELISA. One representative experiment out of three independent ones is shown.

The IL-18 specificity of rBCGhIL-18-driven enhancement of IFN-*γ* production in DC-naive T cell cocultures was demonstrated in the anti-IL-18 neutralizing experiments conducted for three individuals (Figure 8). As shown in Figure 8, in the presence of the human anti-IL-18 antibody, the production of IFN-*γ* by naive T cells was reduced in response to rBCGhIL-18-stimulated DC coincubation, both after 24 and 96 h of culture; this was much less the case for rBCGhIL-18-stimulated DC-memory T cell cultures.

## 4. Discussion

Previous studies in mice have shown that, in response to BCG, IL-18 might favor the Th1 cytokine production. The synergistic effects between IL-18 and BCG in IFN-*γ*-dependent anti-mycobacterial protective immunity have been reported. The use of recombinant BCG producing IL-18 may be an attractive tool to polarize immune responses involved in specific defense mechanisms. Here, we constructed a recombinant BCG strain producing human IL-18 and investigated its effect on DCs and on DC-dependent IFN-*γ* production by CD4^+^ T cells in a human* in vitro* model. Live BCG bacilli producing or not producing human IL-18 were used in the study to stimulate human DCs.

First, we examined the effect of rBCGhIL-18 on the expression of cell surface signal transduction receptors on DCs. IL-18 has been reported to promote the expression of costimulatory and adhesion molecules on the macrophages and DCs [30, 31]. Therefore, we examined the effect of rBCGhIL-18 on the expression of DC surface receptors indispensable in the formation of an immune synapse between DCs and T cells. We found that rBCGh-IL-18 upregulated CD86, and to a lesser extent, CD80, while other DC surface molecules (HLA-DR, CD40) were not upregulated. Nonrecombinant BCG also increased the expression of these receptors on DCs, which is in contrast to the results reported by Manickam and Sivanandham [32] showing reduced expression of CD80 but not CD86 on BCG- or PPD-stimulated DCs from patients with cervical cancer. The reasons for the discrepancy between the two studies are not clear and could be due to different experimental conditions, such as different MOIs used in the two studies and the health status of the DCs donors. DC-SIGN surface expression appeared to be decreased upon incubation of the DCs with PPD, BCG, rBCGhIL-18, or LPS as compared to unstimulated DCs. The role of DC-SIGN as an important host ligand in mycobacterial infection seems to be more complex since several DC-SIGN ligands on* M. bovis *BCG have been described [33]. Some studies have shown that* M. tuberculosis, M. avium *subsp.* paratuberculosis* and* M. bovis* BCG can bind to DC-SIGN to promote entry into human DCs and alveolar macrophages, in which these bacteria can survive [34, 35]. A decrease in DC-SIGN expression upon incubation with BCG may thus have an impact on the mycobacterial uptake and intracellular survival of mycobacteria.

In the second part, the examination of the cytokine profile of stimulated DCs revealed no significant effect of live BCG or rBCGhIL-18 and PPD on IL-12 production, although this cytokine was induced by LPS-stimulated DCs. The lack of IL-12 induction is surprising, as* M. tuberculosis* H37Rv, BCG, and* M. bovis* have been described to induce IL-12 production by murine macrophages and DCs [36, 37]. Other studies have shown that* M. tuberculosis* H37Rv and BCG do not induce IL-12 production in human macrophages and DCs [38, 39]. The inability of BCG or rBCGhIL-18 to induce IL-12 by DCs might be related to the IL-10 induction by these mycobacteria, as endogenous IL-10 was shown to suppress BCG-driven IL-12 production in DCs, and, in addition, downregulates the migration of antigen-loaded DCs to the draining lymph nodes, thereby decreasing antimycobacterial cellular response [40, 41]. It has also been suggested that IL-10 is linked with the ability of* M. tuberculosis* to evade host immune responses and mediates long-term infections [42]. In our experimental conditions, we found that rBCGhIL-18 induced significantly more IL-10 by the DCs compared to nonrecombinant BCG or PPD. Significantly higher levels of IL-10 have also been shown by spleen cells of mice infected with recombinant BCG producing murine IL-18 compared to splenocytes from mice infected with non-recombinant BCG [19].

In contrast to IL-12, DCs stimulated with rBCGhIL-18 produced higher amounts of IL-23, compared to DCs treated with BCG or PPD. IL-23 is a heterodimeric cytokine, which shares the p40 subunit with IL-12, but this subunit is covalently linked to the specific p19 subunit [43, 44]. This cytokine can mediate inflammatory and pathological processes either by activating Th17 cells or by directly stimulating macrophages to secrete IL-1, TNF-*α*, and IL-6 [45, 46]. Initially, overlapping functions of IL-12 and IL-23 were postulated for IFN-*γ* production in PHA blast T cells, as well as in CD45RO^+^ memory T cells [44]. Later studies, both* in vitro* and* in vivo*, revealed that IL-23 may negatively regulate IL-12-induced effector functions. IL-23 strongly reduces IL-12-driven secretion of IFN-*γ* by CD8^+^ T cells, with less prominent effects in NK and CD4^+^ T cells [47]. Zhang et al. [37] showed that* M. bovis* induces preferentially IL-23 rather than IL-12 by murine bone marrow-derived DCs. Our results are consistent with these studies, as we found a slight increase in IL-23 production by BCG-treated DCs compared to control DCs, although this did not reach statistical significance.

Among the chemokines involved in the specific attraction of Th1 cells, CXCL10 (IP-10) was described as the major one [48, 49]. Enhanced production of IP-10 by DCs stimulated with mycobacteria has been reported [50, 51], and IP-10 has even been proposed as a biomarker for* M. tuberculosis* infection [52]. In this study, we found that the production of human IL-18 significantly enhanced the ability of BCG to induce the IP-10 production by DCs, implying that this might lead to enhanced interactions of DCs with the Th1 cell population.

We previously reported that circulating naive CD45RA^+^CD4^+^T cells from BCG-vaccinated volunteers become effector helper cells producing IFN-*γ* upon stimulation by autologous DC pulsed with PPD or infected with live BCG [28].

The present results extend these observations showing significantly enhanced IFN-*γ* production by naive CD4^+^ T cells stimulated by rBCGhIL18-infected DCs. The same DCs induced much weaker production of IFN-*γ* in memory CD4^+^ T cells. However, stimulation of DC with rBCGhIL-18 or nonrecombinant BCG led to similar levels of IFN-*γ* production by memory T cells. The strong induction of IFN-*γ* secretion by naive T cells incubated with rBCGhIL-18-stimulated DC in the absence of detectable amounts of IL-12 may have been due to the presence of IL-18R on the surface of naive T cells. On the other hand, strong IFN-*γ* production may lead to the upregulation of IL-18R on DCs [31], which may result in an optimized cytokine environment for DC interactions with naive CD4^+^ T cells and thus enhance Th1 responses.

In cocultures of naive CD4^+^ T cells and rBCGhIL18-infected DCs a statistically significant enhancement in IL-10 production was noticed. Much lower levels of IL-10 were present in the cocultures of naive T cells with DCs which were previously exposed to BCG or PPD. This observation is in agreement with the results reported by Madura Larsen et al. [39] showing that the interaction between BCG and DCs leads to the development of naive T cells into IL10-producing T cells. In a murine model,* M. bovis*-stimulated DCs also induced high levels of IL-10 by CD4^+^ T cells [37]. The discriminating effects of rBCGhIL18-infected DCs on IL-10 production by naive and memory CD4^+^ T cells, as shown in this study, open potential innovative possibilities for the development of new TB vaccines. In our experimental model recombinant rBCGhIL18-infected DCs preferentially induced IL-10 production in naive CD4^+^ T cells, whereas DCs infected with non-recombinant BCG effectively induced IL-10 production in memory CD4^+^ T cells.

In conclusion, in the present report, we demonstrated a remarkable advantage of recombinant rBCGhIL-18 producing human IL-18 over nonrecombinant BCG in stimulating human DC to preferentially trigger strong IFN-*γ* secretion by naive CD4^+^ T cells, which is accompanied by moderately elevated IL-10 production. Although IFN-*γ* responses are clearly required for protection against TB, recent studies have shown a lack of correlation between the degree of immune protection induced by BCG and IFN-*γ* production by CD4^+^ T cells [53, 54]. In addition, we have recently observed significantly enhanced IFN-*γ* responses to specific antigens of* M. tuberculosis *in patients with active tuberculosis [55]. Thus, antigen-specific IFN-*γ* responses may both play an essential role in protection against mycobacteria and be involved in mycobacteria-driven inflammatory processes. Thus, the concomitant enhancement of IFN-*γ* and IL-10 production in cocultures of rBCGhIL-18 stimulated DCs and naive CD4^+^ T cells might provide balanced immunizing effects. Obviously, more work is required to better understand the relationship between the discriminating activities of recombinant rBCGhIL-18 producing human IL-18.
